# Identification of Tumor Microenvironment-Related Prognostic Biomarkers for Ovarian Serous Cancer 3-Year Mortality Using Targeted Maximum Likelihood Estimation: A TCGA Data Mining Study

**DOI:** 10.3389/fgene.2021.625145

**Published:** 2021-06-03

**Authors:** Lu Wang, Xiaoru Sun, Chuandi Jin, Yue Fan, Fuzhong Xue

**Affiliations:** ^1^Institute for Medical Dataology, Cheeloo College of Medicine, Shandong University, Jinan, China; ^2^Department of Biostatistics, School of Public Health, Cheeloo College of Medicine, Shandong University, Jinan, China; ^3^Key Laboratory of Trace Elements and Endemic Diseases, National Health and Family Planning Commission, School of Public Health, Xi’an Jiaotong University, Xi’an, China

**Keywords:** targeted maximum likelihood estimation, ovarian cancer, tumor microenvironment, gene expression, prognostic biomarker

## Abstract

Ovarian serous cancer (OSC) is one of the leading causes of death across the world. The role of the tumor microenvironment (TME) in OSC has received increasing attention. Targeted maximum likelihood estimation (TMLE) is developed under a counterfactual framework to produce effect estimation for both the population level and individual level. In this study, we aim to identify TME-related genes and using the TMLE method to estimate their effects on the 3-year mortality of OSC. In total, 285 OSC patients from the TCGA database constituted the studying population. ESTIMATE algorithm was implemented to evaluate immune and stromal components in TME. Differential analysis between high-score and low-score groups regarding ImmuneScore and StromalScore was performed to select shared differential expressed genes (DEGs). Univariate logistic regression analysis was followed to evaluate associations between DEGs and clinical pathologic factors with 3-year mortality. TMLE analysis was conducted to estimate the average effect (AE), individual effect (IE), and marginal odds ratio (MOR). The validation was performed using three datasets from Gene Expression Omnibus (GEO) database. Additionally, 355 DEGs were selected after differential analysis, and 12 genes from DEGs were significant after univariate logistic regression. Four genes remained significant after TMLE analysis. In specific, ARID3C and FREM2 were negatively correlated with OSC 3-year mortality. CROCC2 and PTF1A were positively correlated with OSC 3-year mortality. Combining of ESTIMATE algorithm and TMLE algorithm, we identified four TME-related genes in OSC. AEs were estimated to provide averaged effects based on the population level, while IEs were estimated to provide individualized effects and may be helpful for precision medicine.

## Introduction

Ovarian cancer is one of the most common cancers and the leading cause of death of all gynecological cancers among women across the world (Bray et al., 2018). Ovarian serous cancer (OSC) is the most common histologic subtype, and it accounts for about 90% of all ovarian tumors (Bell et al., 2011). In the United States, approximately 1 in 78 women will develop ovarian cancer in their lifetime while the all-stage 5-year survival rate of ovarian cancers is only 47% (Torre et al., 2018). Over 70% of ovarian cancers are diagnosed at advanced stages (stage III or IV) and usually exhibit poor prognosis with a 5-year overall survival rate of ∼30% (Howlader et al., 2016; Torre et al., 2018). The high mortality may partly due to the non-specific symptoms, lack of specific screening tools, and non-specific clinical drugs (Diaz-Padilla et al., 2012). Therefore, the identification of biomarkers with a poor short time prognosis remains significant. In this study, we focused on OSC 3-year mortality to evaluate short survival time-related biomarkers.

The role of the tumor microenvironment (TME) in OSC has been discussed increasing in recent years. TME refers to the constitution of a complex network that includes different subsets of stromal cells, immune cells, fibroblasts, blood vessels, extracellular matrix (ECM), endothelial cell precursors, and secreted factors (Kenny et al., 2007). It is now established that TME plays an important role in cancer progression, metastasis, and resistance to the therapies through complex interactions (Luo et al., 2016; Sung et al., 2016; Wang et al., 2017; Finkernagel et al., 2019). In particular, a positive correlation was observed between cancer-associated fibroblasts (CAFs) and the clinical stage. The co-culture of CAFs with ovarian cancer cells further showed stimulation of cancer cell invasion and migration (Zhang et al., 2011). Moreover, biomarkers like CD163, CD206/CD68 ratio, B7-H4, and IL-10 produced by tumor-associated macrophages (TAMs) were associated with survival and clinical outcome of ovarian cancer patients (Bartlett et al., 2001; Lan et al., 2013). However, it may still lack a full understanding of the molecular interactions between immune and stromal components in TME and how they affect ovarian cancer progression. Therefore, a further and deeper understanding of TME is urgently needed to better understand how their stromal and immune components affect OSC survival and clinical outcome.

To identify such relationships, classic epidemiologic analysis such as the regression method has discovered numerous biomarkers. However, those associations can be biased by confounding factors or misspecification of a parametric outcome model. Targeted maximum likelihood estimation (TMLE) is an efficient, double robust, semi-parametric methodology that has been proposed for estimating marginal effects (van der Laan and Rubin, 2006; van der Laan et al., 2009). TMLE is developed under the causal inference framework and allows researchers to estimate the average effect (AE) for binary treatment (Rubin, 1974; Greenland and Robins, 1986; Luque-Fernandez et al., 2018). Though TMLE usually shows advantages over the commonly used propensity score method or G-computation with both point and interval estimation, it has not been widely implemented in epidemiologic research including biomarkers discovering and omics data mining.

In this study, we aimed to discover TME associated biomarkers that have effects on the 3-year mortality of OSC patients. OSC patients were collected from The Cancer Genome Atlas (TCGA) database with RNA-sequencing data employed for gene expression data. We present our study using the ESTIMATE algorithm to access the immune and stromal components level in ovarian tumor samples. Differential expression genes possibly representing TME status were selected. Univariate analysis followed by covariates selection analysis was performed to determine candidate prognostic biomarkers and their confounding factors sets. The TMLE method was conducted to evaluate average and individual effects, respectively. Four genes, ARID3C, CROCC2, FREM2, and PTF1A, were successfully selected as potential OSC 3-year mortality-related prognostic biomarkers.

## Results

### Clinical Characteristics of the Study Patients

**FIGURE 1:**
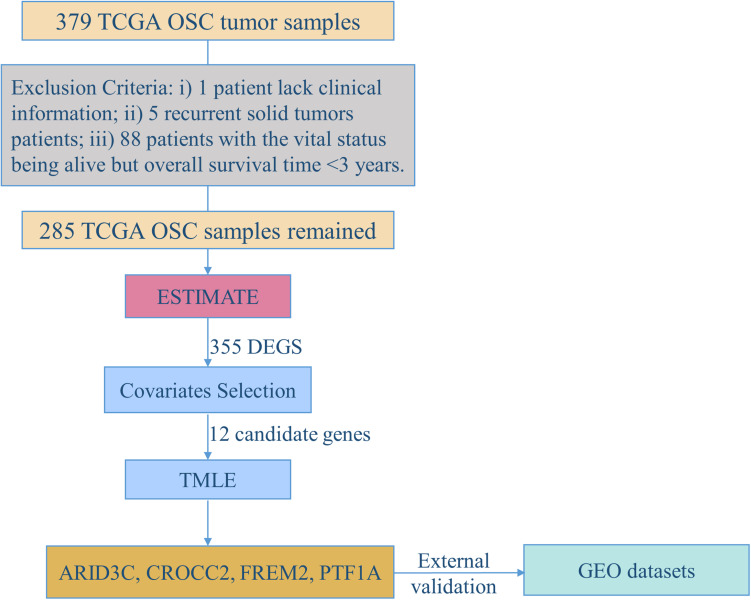
The flowchart for identification of prognostic biomarkers.

Figure 1 demonstrates the flowchart for the identification of prognostic biomarkers. 285 OSC patients from the TCGA database were included for statistical analysis. The detailed clinical characteristics of the studying population were summarized in Table 1.

**TABLE 1:** Description of clinic pathologic factors for 285 ovarian cancer patients from TCGA.

Clinic pathologic variable*	**Dead (*N* = 115)**	**Alive (*N* = 170)**
Age (years)	63.89 (11.26)	59.03 (10.2)
**FIGO Stage**		
I	1 (0.87)	0 (0.00)
II	2 (1.74)	10 (5.88)
III	88 (76.52)	135 (79.41)
IV	23 (20.00)	25 (14.71)
Unknown	1 (0.87)	0 (0.00)
**Grade**		
GB	1 (0.87)	1 (0.59)
G2	8 (6.96)	25 (14.71)
G3	104 (90.43)	140 (82.35)
G4	0 (0.00)	1 (0.59)
Unknown	2 (1.74)	3 (1.76)
**Venous invasion**		
NO	9 (7.83)	17 (10.00)
YES	17 (14.78)	24 (14.12)
Unknown	89 (77.39)	129 (75.88)
**Lymphatic invasion**		
NO	9 (7.83)	19 (11.18)
YES	32 (27.83)	33 (19.41)
Unknown	74 (64.35)	118 (69.41)
**Tumor residual disease**		
No macroscopic disease	11 (9.57)	27 (15.88)
1–10 mm	53 (46.09)	85 (50.00)
11–20 mm	8 (6.96)	12 (7.06)
>20 mm	32 (27.83)	25 (14.71)
Unknown	11 (9.57)	21 (12.35)

*Data are mean ± SD and frequency (percent) for numeric and category variables, respectively.

### DEGs Shared by ImmuneScore and StromalScore in OSC

**FIGURE 2:**
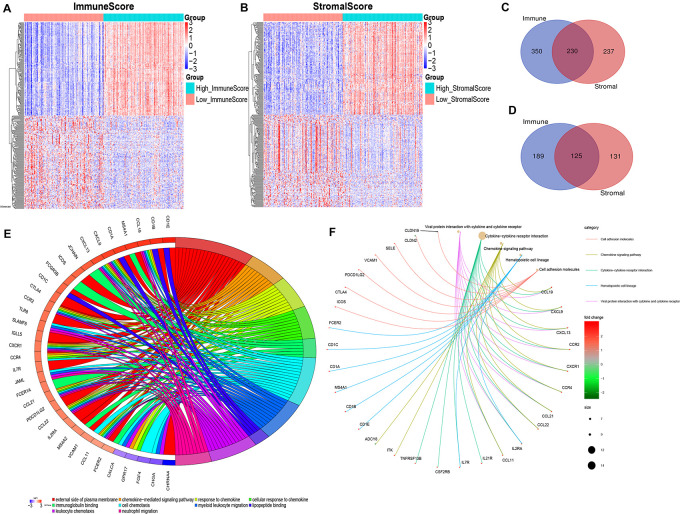
Identification and functional enrichment of differentially expressed genes. **(A)** Heatmap for DEGs generated by comparison of the high ImmuneScore group with the low ImmuneScore group. Rows and columns represent DEGs and samples, respectively. Significant DEGs were determined with FDR < 0.05 and | fold-change| > 1.5 after log2 transformation. **(B)** Heatmap for DEGs in StromalScore, similar with **(A)**. **(C,D)** Venn plots showing common upregulated and downregulated DEGs shared by ImmuneScore and StromalScore. **(E)** GOChord plot of GO enrichment analysis for common DEGs showing enriched GO terms and corresponding genes. The significance level was set to be with both FDR and *q* < 0.05. **(F)** Cnetplot of KEGG enrichment analysis for common DEGs showing enriched KEGG terms and corresponding genes. The significance level was set to be with both FDR and *q* < 0.05.

By comparing the gene expression profiles of patients with high immune scores against those with low immune scores, a total of 894 (580 upregulated and 314 downregulated) DEGs were identified (Figures 2A,C,D). A total of 723 (467 upregulated and 256 downregulated) DEGs were identified by comparing the high and low stromal score groups (Figures 2B–D). | Log2 (fold-change)| > 1.5 and FDR < 0.05 were used as criterions for screening DEGs. A total of 230 DEGs were in common among the high immune/stromal score groups. A total of 125 DEGs were in common among the low immune/stromal score groups. These DEGs (total 355 genes) were possibly determinate factors for the status of TME. Results from gene ontology (GO) enrichment analysis indicated that the DEGs were mainly enriched for the immune-related GO terms, such as chemokine signaling pathway and immunoglobulin binding (Figure 2E). Based on the Kyoto Encyclopedia of Genes and Genomes (KEGG) enrichment analysis, chemokine signaling pathway, cytokine–cytokine receptor interaction, and hematopoietic cell lineage were significantly enriched pathways (Figure 2F). In all, these DEGs mainly mapped to immune-related functions and therefore indicate that immune components as the leading feature of TME in OSC.

### Selection of Candidate Potential Prognostic Biomarkers and Confounding Factors

In the process of screening for prognostic-related biomarkers, the 355 DEGs shared by the high immune/stromal score and low immune/stromal score groups together with clinical variables were subjected to a univariate analysis. Out of these, 12 DEGs, age, and tumor residual disease were found to be significantly (with *p*-value < 0.05) correlated with the 3-year mortality of OSC patients from the TCGA database. The detailed information regarding the associations of univariate analysis is provided in Table 2. To determine the proper covariates set to control in the following TMLE analysis, each one of 12 candidate markers was subsequently undergoing a covariates selection procedure. Supplementary Figure 1 shows the covariates set and their correlations for 12 candidate potential prognostic biomarkers. The number of confounding factors for each gene varied from 7 to 13, with FREM2 had maximum covariates set while SOHLH1 owning minimum covariates set. Age was the common confounding factor shared by all 12 candidate genes, indicating the essential role of age as a confounder. Besides, FREM2 had maximum confounders as well as the common confounder shared by the other 11 candidate genes, indicating that FREM2 may be a hub gene in the OSC prognosis regulation network.

**TABLE 2:** Univariate logistic regression showing significant associations with 3-year mortality of OSC.

Variable*	**Levels**	**OR/Coefficient**	**95% CI**	***P*-value**
IGLV5-37	High expression level	1.82	(1.13; 2.93)	0.014
IGHJ3	High expression level	1.63	(1.01; 2.63)	0.046
IGHV1OR15-1	High expression level	1.82	(1.05; 3.15)	0.033
PTF1A	High expression level	1.73	(1.06; 2.84)	0.029
ARID3C	High expression level	0.59	(0.37; 0.96)	0.032
FREM2	High expression level	0.61	(0.38; 0.99)	0.046
CLDN19	High expression level	1.65	(1.02; 2.65)	0.040
CROCC2	High expression level	1.65	(1.02; 2.65)	0.040
SPAG6	High expression level	1.73	(1.07; 2.79)	0.025
MAGEA9B	High expression level	1.97	(1.13; 3.41)	0.016
ONECUT1	High expression level	1.71	(1.01; 2.91)	0.046
SOHLH1	High expression level	1.67	(1.03; 2.69)	0.036
Tumor residual disease	No macroscopic disease	Ref	–	–
	1–10 mm	1.53	(0.70; 3.34)	0.285
	11–20 mm	1.64	(0.53; 5.10)	0.396
	>20 mm	3.14	(1.31; 7.54)	0.010
	Unknown	1.29	(0.47; 3.54)	0.626
Age	year	0.01	(0.00; 0.01)	<0.001

*Data are odds ratio and 95% confidence interval for category variables as well as coefficient (β) and 95% confidence interval for the numeric variable (Age).

### TMLE Estimation

**FIGURE 3:**
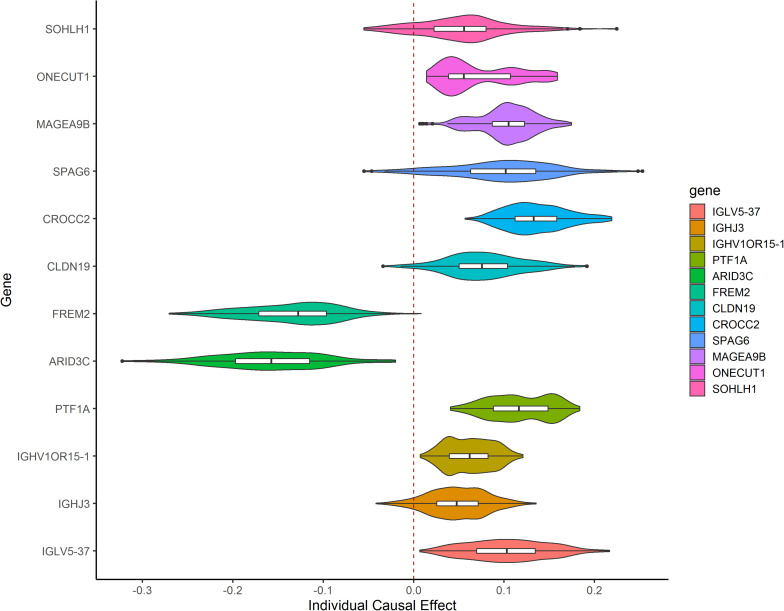
Violin plot showing IE estimates the distribution of 12 candidate genes. IE was calculated as the marginal risk difference of 3-year mortality for each OSC patient with high gene expression versus low gene expression.

After TMLE analysis, the association of four genes (ARID3C, CROCC2, FREM2, and PTF1A) with OSC 3-year mortality remained significant (*p* < 0.05). Their marginal odds ratio (MOR), AE, and 95% CI estimates are shown in Table 3. TMLE analysis suggesting positive associations between a high expression level of ARID3C and FREM2 with promising prognosis and negative associations for CROCC2 and PTF1A. The Violin plot of individual effect (IE) estimates for all candidate genes was shown in Figure 3. The meaning of IE is different from AE for the former one representing individualized effect estimates and the minority may have opposing effects compare with the average effect. Those inconsistent was observed in the IE of FREM2, indicating that the effect of this gene may differ in different population subsets. Compared with AE, IE may be more meaningful in terms of personalized treatment for precision medicine.

**TABLE 3:** Four genes shown significant association after TMLE analysis.

**Gene**	**Chromosome**	**MOR**	**95% CI**	***P*-MOR**	**AE**	**95% CI**	***P*-AE**
ARID3C	9	0.51	(0.32, 0.82)	0.005	−0.16	(−0.27, −0.05)	0.004
CROCC2	2	1.76	(1.12, 2.78)	0.015	0.14	(0.03, 0.24)	0.014
FREM2	13	0.57	(0.36, 0.90)	0.016	−0.14	(−0.24, −0.03)	0.015
PTF1A	10	1.63	(1.02, 2.60)	0.042	0.12	(0.00, 0.23)	0.043

### Validation in the Gene Expression Omnibus (GEO) Dataset

**FIGURE 4:**
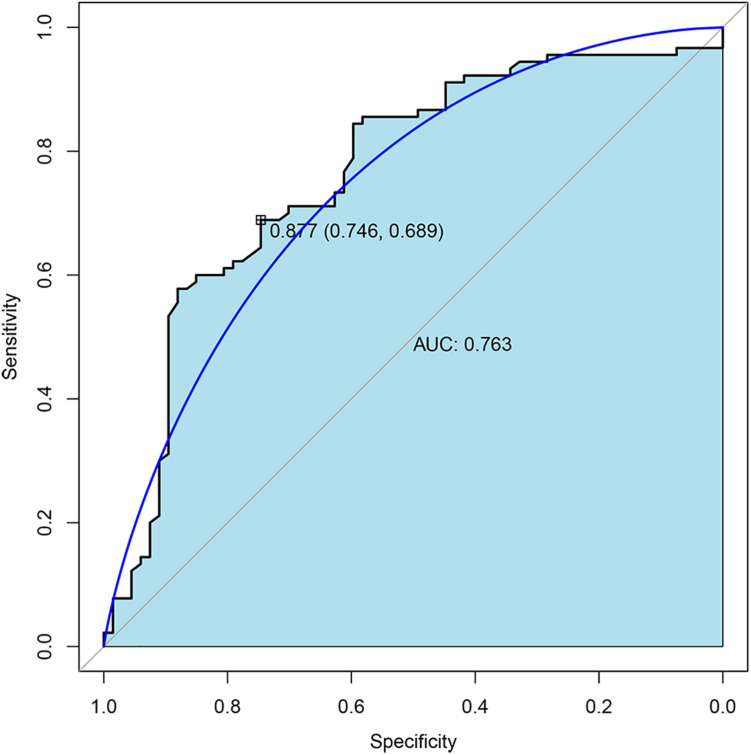
Receiver operating characteristic (ROC) curves and area under the curve (AUC) of the combination of four gene biomarkers for 3-year mortality prediction of OSC patients in GEO dataset.

To further validate the expression of four TMLE significant genes (ARID3C, CROCC2, FREM2, and PTF1A), we conducted external validation using Gene Expression Omnibus (GEO) database. Due to the fact that GEO datasets contain limited gene expression profiles, three GEO datasets (GSE53963, GSE26193, and GSE13876) were obtained and successfully validated the expression of three genes (PTF1A, FREM2, and CROCC) separately (Supplementary Table 1). The expression of two genes (CROCC and PTF1A) were upregulated while FREM2 was downregulated in OSC patients with poor prognosis compared to promising prognosis. These results were largely consistent with our results in TCGA data. The sensitivity and specificity of each verified gene were evaluated. The sensitivity and specificity of PTF1A calculated from the GSE53963 dataset were 0.74 and 0.56, respectively. The sensitivity and specificity of FREM2 calculated from the GSE26193 dataset were 0.61 and 0.62, respectively. The sensitivity and specificity of CROCC calculated from the GSE13876 dataset were 0.59 and 0.63, respectively. The receiver operating characteristic (ROC) curve analyses and the area under the curve (AUC) were used to assess the discriminatory ability of four genes (ARID3C, CROCC, FREM2, and PTF1A) among 67 OSC patients with promising prognosis and 90 patients with poor prognosis derived from GSE13876 dataset. The AUC of all four genes was 0.763 (Figure 4). The specificity and sensitivity were 0.746 and 0.689, respectively.

## Discussion

In the presented study, we attempted to identify TME-related genes that have an effect on the 3-year mortality of OSC patients from the TCGA database. TMLE algorithm was performed to derive AE, IE, and MOR with doubly robust to model misspecification. ARID3C, CROCC2, FREM2, and PTF1A were identified as prognostic biomarkers for OSC patients. Two of them (FREM2 and PTF1A), alongside CROCC, were successfully validated in three GEO datasets. In addition to averaged effects estimation, individualized effects were estimated for each patient. Functional analysis indicated that ARID3C, CROCC2, and FREM2 might be involved in the immune status of TME.

In our research, increased expression of AT-rich interaction domain 3C (ARID3C) was found to be significantly associated with better OSC prognosis. ARID3C was recently characterized and belongs to the AT-rich interaction domain (ARID) family of proteins (Tidwell et al., 2011). The ARID3 subfamily consists of 3 members ARID3A, B, and C, which are expressed throughout most of hematopoietic development (Heng et al., 2008). The expression of ARID3A, the founding member of the subfamily, is tightly regulated during B cell differentiation (Webb et al., 1998; Heng et al., 2008). A recent study demonstrated that ARID3B also plays an important role in normal B cell development (Kurkewich et al., 2016). Similarly, ARID3C transcripts and proteins are expressed especially within B lineage lymphocytes and significantly co-activates ARID3A-dependent immunoglobulin heavy chain gene (IgH) transcription (Tidwell et al., 2011). In recent research, high IgH mRNA levels were reported positively associated with improved prognosis of breast cancer (Larsson et al., 2020). Indicating that ARID3C could modulate IgH activity to enhance immune response and improve the prognosis of cancers. The function of B lymphocytes in tumor development has contradictory opinions. Their presence has been found to be associated with a good prognosis in ovarian cancer through the priming of CD4+ and CD8+ T cells and the production of tumor-specific IgGs (Stumpf et al., 2009; Nielsen et al., 2012). Conversely, high B cell infiltration negatively correlates with patient survival in ovarian cancer (Gentles et al., 2015). In addition, ARID3A and ARID3B, two closely related paralogs of ARID3C, are reported to be important associated with tumorigenesis (Samyesudhas et al., 2014; Chien et al., 2015; Nakahara et al., 2017; Tu et al., 2019; Dausinas et al., 2020).

The FRAS1-related extracellular matrix 2 (FREM2) is an extracellular matrix protein that localizes in the lamina densa of epithelial basement membranes (Kiyozumi et al., 2006; Chiotaki et al., 2007). In the extracellular matrix (ECM), FREM2, and FRAS1 form a self-stabilizing complex with FREM1, which plays an important role in cell adhesion and intercellular signaling (Kiyozumi et al., 2006). ECM, as important content of TME, provides structural support as well as biochemical and biomechanical cues for cancer cell growth (De Palma et al., 2017). Previous studies have reported that mutations in this gene are associated with Fraser syndrome and play an important role in various tumorigenesis. In glioblastomas, FREM2 expression is positively associated with a favorable prognosis of IDH-WT glioblastomas, which is consistent with our research result (Jovčevska et al., 2019). In ovarian cancer, the differential expression of FREM2 has been reported in a few studies (Dakubo, 2019; Zhu et al., 2020). FREM2 has been identified as a target gene of transcription factor AP-2 gamma (TFAP2C, AP-2γ) (Woodfield et al., 2010), which is a sequence-specific DNA-binding transcription factor and belongs to the AP-2 family. AP-2γ has been established as a prognostic factor in human breast cancer (Cyr et al., 2015). Its paralog, AP-2α, has been observed to play a tumor-suppressive role in ovarian cancer (Sumigama et al., 2004).

Pancreas-associated transcription factor 1a (PTF1A) plays a critical role in controlling the development and physiological function of many organs, including the pancreas, brain, spinal cord, retina, and others (Jin and Xiang, 2019). Although there are limited studies about PTF1A outside pancreas, PTF1A protein abundance is highest in the ovary at the tissue level, indicating that it may have an important role in the ovary (Jin and Xiang, 2019). Furthermore, PTF1A is involved in the Notch-mediated HES/HEY network, which has been found by many studies being activated in ovarian cancer (Bell et al., 2011; Bocchicchio et al., 2019; Huang et al., 2019; Hubbard et al., 2019; Eoh et al., 2020). The Notch signaling has been found to be able to ensure the proliferation and migration of ovarian cancer cells and therefore controls ovarian cancer cell survival (Bocchicchio et al., 2019). In addition, we found that one study showed that T follicular helper (Tfh) cell generation and function are reliant on Notch signaling (Dell’aringa and Lee Reinhardt, 2018).

CROCC2 is a newly annotated protein in Gascoigne et al. (2012) that has similarity to CROCC and belongs to the ciliary rootlet coiled-coil rootletin family. Rootletin is a structural component of the ciliary rootlet (Yang et al., 2002) and primary cilia that has been suggested to have an important role in carcinogenesis through facilitating cell signaling pathways platelet-derived growth factor receptor-a and Wnt pathways that are commonly dysregulated in many tumors (Yasar et al., 2017). Our study suggesting a positive effect between high expression of CROCC2 with poor 3-year OSC mortality. Further studies should focus on the biological function of CROCC2 on the prognosis of ovarian cancer.

To our knowledge, our work is the first to use the ESTIMATE algorithm combined with TMLE methodology to explore molecular markers that have effects on OSC 3-year prognosis. The covariate selection procedure makes sure appropriate confounding factors are selected to reduce bias or loss of power. The prognostic biomarkers were driven from a causal inference framework-based TMLE algorithm. Such methodologies can be used to better inform future clinical therapy. Individual effect estimates were provided to display personal differences and may be helpful for precision medicine. There are also limitations to this study. To gain explainable effect estimation, we dichotomized the exposure (gene expression) and define the 3-year mortality of OSC instead of overall survival as the outcome variable. Therefore, we lost 88 samples due to the missing outcome variable. Further studies can be focused on the biological function role of identified genes in the network of TME.

## Materials and Methods

### Data Source and Outcome Definition

Transcriptome RNA-seq read counts data of 379 OSC samples and the corresponding clinical data were downloaded from the TCGA database^1^ using the R package “TCGAbiolinks” (Colaprico et al., 2016). The mRNA data was already processed using the GDC mRNA quantification analysis pipeline, which measures gene-level expression in HT-Seq raw read count. These values are generated through this pipeline by first aligning reads to the GRCh38 reference genome and then by quantifying the mapped reads. More information on the GDC pipeline can be found at: https://docs.gdc.cancer.gov/Data/Bioinformatics_Pipelines/Expression_mRNA_Pipeline/. Gene mapping was performed using the gencode v22 annotation file. The samples were trimmed to 378 to include only patients with corresponding clinical information. The data from 373 primary solid tumors patients were further retained after removing five recurrent solid tumor samples. Finally, 88 samples were excluded with the vital status being alive but overall survival time <3 years. In total, 285 OSC patients were kept after quality control and were included in the following analysis. The outcome binary was defined as 3-year mortality of OSC, alive status was defined as patients with OS time ≥ 3 years, and dead status was defined as patients with the vital status being dead and OS time <3 years.

### TME Construction

ESTIMATE algorithm was performed to estimate immune and stromal components in TME for each sample using the R package “estimate” (Yoshihara et al., 2013). The ImmuneScore, StromalScore, and ESTIMATEScore were calculated corresponding to the level of immune cells, stromal cells, and the sum of both, respectively. A higher ESTIMATEScore, StromalScore, and ImmuneScore, respectively, represent the lower tumor purity and higher infiltration levels of stromal and immune cells in tumor tissue.

### Differentiation Analysis Between High-Score and Low-Score Groups Regarding ImmuneScore and StromalScore

To better understand the correlation between gene expression profiles and immune and/or stromal scores, patients were divided into two groups based on the median value of the ImmuneScore and StromalScore, respectively. R package “DESeq2” was used to perform differentiation analysis. Genes where all counts were less than half sample numbers were filtered. The differentiation analysis was conducted in the high immune score group vs. the low immune score group and the high stromal score group vs. the low stromal score group, separately. For each DE analysis, we specified | log2FC(fold-change)| > 1.5 (high immune/stromal score group vs. low immune/stromal score group) and false discovery rate (FDR) <0.05 as cutoffs to identify significant DEGs. The qualified DEGs that both upregulated immune and stromal scores or downregulated immune and stromal scores were finally selected for further analysis.

### GO and KEGG Enrichment Analysis

For shared DEGs between high ImmuneScore and low ImmuneScore as well as high StromalScore and low StromalScore, GO and KEGG pathway enrichment analyses were conducted to investigate the shared biological function by using R packages “clusterProfiler,” “enrichplot,” “GOplot,” and “ggplot2.” Only terms with both FDR and *q*-value of <0.05 were considered significantly enriched.

### Univariate Analysis of Associated Genes and Clinical Pathologic Factors

In the presented study, all OSC samples were grouped into a high-expression group and low-expression group compared with the corresponding median expression level for each DEG. Univariate analysis of the association of binary DEGs and other clinical pathologic factors with 3-year mortality was evaluated using logistic regression. Statistical significance was defined as *p* < 0.05. For factors significantly associated with 3-year mortality in univariate analyses, a covariates selection procedure was conducted.

### Selection of the Minimal Sets of Confounding Covariates

It is crucial to select a proper set of covariates for estimating unbiased effects between a treatment effect and an outcome variable in observational studies. Including unnecessary covariates may result in a loss of power or biased variance while lack of covariates may lead to unadjusted confounding effects. De Luna, Waernbaum, and Richardson (Luna et al., 2011) proposed a data-driven algorithm for the selection of minimal sets of covariates. Let *G* = {*G*_1_,*G*_2_,…,*G*_*p*_} denote *p* binary candidate potential prognostic biomarkers, *X* = {*X*_1_,*X*_2_,…,*X*_*m*_} denote *m* selected clinical covariates and *Y* denote OSC 3-year mortality. Then *C* = {(*G*\*G*_*j*_)+*X*} denotes the complete covariate set for gene *G*_*j*_ and *Y*. We aimed to determine a minimum of confounding covariates of set *W* that satisfy 1) *Y⊥C| W* and 2) *G_*j*_⊥C| W*. To do so, we first found *V* to be a minimal set of *C*, making *Y* and the covariates not included in *V* conditionally independent, *Y⊥C\V| V*. Then, W is determined as the minimal set of V, which means the *G*_*j*_ and the covariates are not included in the *W* that is conditionally independent: *G_*j*_⊥V\W| W*. R package *CovSel* was performed to select the minimal covariate sets *V* for each *G*_*j*_.

### TMLE Estimation Method

In this study, we aimed to estimate the effects of gene expression levels on OSC 3-year mortality. We are interested in three estimates of the effects: the AE, interpreted as the marginal risk difference of 3-year mortality for OSC patients with high gene expression versus low gene expression compared to median expression level; the IE, defined as the marginal risk difference of 3-year mortality for each OSC patient with high gene expression versus low gene expression; and the MOR, interpreted as the odds ratio of death for patients with high gene expression versus low gene expression. Effects formalized by Rubin (1974) were developed under the potential outcome framework, and the casual parameters are therefore defined as the following:

(1) AE=E⁢[Y⁢(1)-Y⁢(0)]

(2) IE=Y⁢(1)-Y⁢(0)

(3) MOR={E⁢[Y⁢(1)]×E⁢[1-Y⁢(0)]}/{E⁢[1-Y⁢(1)]×E⁢[Y⁢(0)]}

where *Y*(1) represents OSC 3-year potential outcome they would have received had they been exposed to high gene expression level compared with median expression level (*G* = 1); and *Y*(0) denotes OSC 3-year potential outcome had they been unexposed. In our study, OSC 3-year outcome *Y* has two possible values: 1 denotes death while 0 denotes alive.

### Identification of the Effect Estimates

For the effect estimates to have a theoretical interpretation, several key assumptions are required: (1) there is the stable unit treatment value assumption (SUTVA), which assumes that the gene expression level of a given individual does not affect the potential OSC 3-year mortality of any other individuals (i.e., non-interference) and that the exposure level is the same for all individuals who were exposed at that level; (2) there are no unmeasured confounders, and this is formalized as (*Y*(1), *Y*(0))⊥*G*| *W*, meaning that the gene expression level and potential mortality outcomes are independent after conditioning on the set of covariates (this assumption cannot be tested using the observed data); and (3) there is positivity, which requires that within strata of *G*, every individual has a non-zero probability of receiving either exposure condition; this is formalized as 0 < P(*G* = 1| *W*) < 1 for a binary exposure. If the positivity assumption is violated, effects will not be identifiable.

Under these assumptions, the effect estimates from Eqn. (1–3) can be expressed as follows:

(4) $$\mathrm{AE}=E_{W}\left[E\left(Y|G=1,W\right)-E\left(Y|G=0,W\right)\right]$$

(5) $${\mathrm{IE}}_{i}=E\left(Y|G=1,W_{i}\right)-E\left(Y|G=0,W_{i}\right)$$

(6) $$M\,O\,R=\frac{E_{W}\left[E\left(Y|G=1,W\right)\right]\times \left\{1-E_{W}\left[E\left(Y|G=0,W\right)\right]\right\}}{\left\{1-E_{W}\left[E\left(Y|G=1,W\right)\right]\right\}\times E_{W}\left[E\left(Y|G=0,W\right)\right]}$$

where IE_*i*_ refers to the individual effect for the *i*-th individual, and W*_*i*_* denotes the *i*-th individual’s vector of covariates.

### Estimation of Effect Estimates

In this article, the TMLE method was implemented to detect the potential prognostic biomarkers of OSC 3-year mortality and estimate their effects, including AE, IE and MOR. Note that, we estimate ATE and MOR from a population level to gain averaged effect and IE from individual level to demonstrate personalized information. In specific, effect estimation with TMLE begins with initial estimates of E(*Y*| *G*, *W*) and P(*G* = 1| *W*), then the following ‘‘targeting’’ step would optimize the bias-variance tradeoff for above estimates. Finally, the updated estimation of E(*Y*| *G*, *W*) is used to generate above effect estimates. To avoid model misspecification and improve the robustness, Super Learner algorithm was constructed for parameter estimation. Five models (glm, glm.interaction, glmnet, xgboost, and randomforest) were selected in this study. R package ‘‘tmle,’’ ‘‘randomForest,’’ ‘‘glmnet,’’ and ‘‘xgboost’’ were performed. All statistical analyses were performed using software R version 3.6.1 from CRAN^2^ and *p* < 0.05 was considered statistically significant.

### Validation in the Gene Expression Omnibus (GEO) Dataset

For the verification of TMLE significant genes, the GEO database was searched. Due to different experiment types and platforms, a single GEO dataset does not generally contain all TMLE significant genes. Hence, three ovarian cancer datasets were chosen, GSE53963 (*n* = 174), GSE26193 (*n* = 107) and GSE13876 (*n* = 157) as validation sets. Data of GSE53963 was obtained by GPL6480 Agilent-014850 Whole Human Genome Microarray 4x44K G4112F (Probe Name version). Data of GSE26193 was obtained by GPL570 [HG-U133_Plus_2] Affymetrix Human Genome U133 Plus 2.0 Array. Data of GSE13876 was obtained by GPL7759 Operon human v3 ∼35K 70-mer two-color oligonucleotide microarrays. All three datasets include complete survival information. The overlapped genes with 355 DEGs were first extracted from GEO datasets. The expression profile of CROCC2 was not available for most GEO datasets. We considered its family member CROCC as the replacement. We then used the average value when a duplicate sample was found and discretized the gene expression data according to the median value. Further, univariate logistic regression was performed between binary DEGs and other clinical pathologic factors with 3-year mortality. Covariates selection and TMLE analysis were followed among DEGs that were significant in univariate analyses. Sensitivity and specificity were calculated for verified TMLE significant genes separately. ROC curve analyses and AUC were used to assess the discriminatory ability of four genes (ARID3C, CROCC, FREM2, and PTF1A) using the GSE13876 dataset. Sensitivity and specificity were calculated for the combination of four genes.

## Conclusion

In summary, with a combination of the ESTIMATE and TMLE algorithms, we identified four TME-related genes in OSC using the TCGA database. With the help of TMLE, effects were estimated for gene expression level on OSC prognosis. ARID3C and FREM2 were potential prognostic biomarkers for promising 3-year survival while CROCC2 and PTF1A might be biomarkers for poor 3-year survival. More importantly, in addition to AE, IE was estimated and provided to present a personalized effect on recognized genes, which may be useful for predicting individual therapy effects. Therefore, further investigation should be conducted to clarify the biological role in metastasis or recurrence of OSC.

## Data Availability Statement

Publicly available datasets were analyzed in this study. This data can be found here: https://portal.gdc.cancer.gov; https://www.ncbi.nlm.nih.gov/geo/.

## Author Contributions

LW and XS performed most of the analyses and wrote the manuscript. FX conceived and designed the study. CJ and YF performed some of the analyses and edited the manuscript. All authors contributed to the article and approved the submitted version.

## Conflict of Interest

The authors declare that the research was conducted in the absence of any commercial or financial relationships that could be construed as a potential conflict of interest.

## Footnotes

1 https://portal.gdc.cancer.gov/

2 http://cran.r-project.org/

## Supplementary Material

Supplementary Figure 1 Heat map showing the correlation between 12 candidates gene and 14 confounding variables. The color shade of each color box represented the corresponding Pearson coefficient value between two variables. For each gene, the black point labeled confounding variables were selected confounders.

Supplementary Table 1 Three genes showed significant association after GEO validation.
