# Supplementary figures and images for: Identification of Tumor Microenvironment-Related Prognostic Biomarkers for Ovarian Serous Cancer 3-Year Mortality Using Targeted Maximum Likelihood Estimation: A TCGA Data Mining Study
Source: Front Genet. 2021 Jun 3;12:625145. doi: 10.3389/fgene.2021.625145 (PMC8211425; doi:10.3389/fgene.2021.625145)

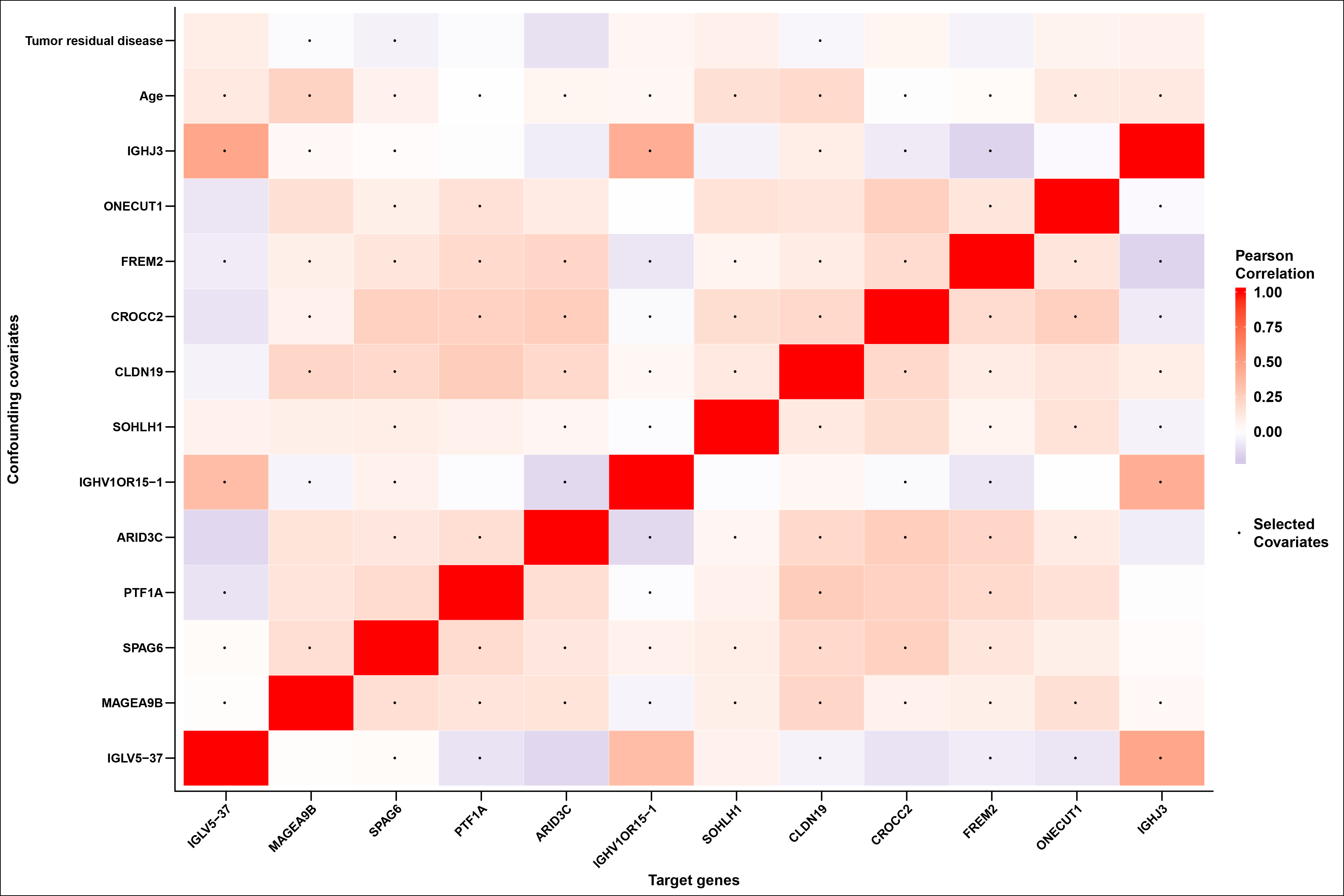

Supplement: Supplementary Figure 1 — Heat map showing the correlation between 12 candidates gene and 14 confounding variables. The color shade of each color box represented the corresponding Pearson coefficient value between two variables. For each gene, the black point labeled confounding variables were selected confounders. [file Image_1.TIF]
