# Supplementary material for: Identification of Tumor Microenvironment-Related Prognostic Biomarkers for Ovarian Serous Cancer 3-Year Mortality Using Targeted Maximum Likelihood Estimation: A TCGA Data Mining Study
Source: Front Genet. 2021 Jun 3;12:625145. doi: 10.3389/fgene.2021.625145 (PMC8211425; doi:10.3389/fgene.2021.625145)
Supplement: Supplementary Table 1 — Three genes showed significant association after GEO validation. [file Table_1.docx]

**Table S1. Three genes shown significant association after GEO validation**

| Gene | GEO dataset | MOR | 95%CI | *P*-MOR | AE | 95%CI | *P*-AE |
| --- | --- | --- | --- | --- | --- | --- | --- |
| PTF1A | GSE53963 | 1.78 | (1.00,3.15) | 0.049 | 0.14 | ( 0.00, 0.28) | 0.046 |
| FREM2 | GSE26193 | 0.43 | (0.20,0.94) | 0.034 | -0.21 | (-0.39,-0.02) | 0.029 |
| CROCC | GSE13876 | 2.35 | (1.30,4.37) | 0.007 | 0.21 | ( 0.06, 0.35) | 0.005 |
